# Antegrade revascularization of the three mesenteric vessels to treat chronic mesenteric ischemia

**DOI:** 10.1093/jscr/rjab328

**Published:** 2021-08-04

**Authors:** Nazih Chaouch, Mohammad M Zagzoog

**Affiliations:** Service of Vascular and Endovascular Surgery, Rene Dubos Hospital Center, Pontoise, France; Service of Vascular and Endovascular Surgery, University of Sorbonne, Paris, France

**Keywords:** chronic mesenteric ischemia, celiac trunk, superior mesenteric artery, and inferior mesenteric artery, occlusion, open surgical revascularization

## Abstract

Chronic mesenteric ischemia is a serious vascular disease that progresses with acute mesenteric ischemia, along with high mortality. Elective intervention has been shown to prevent this progression and improve symptoms. Controversy remains about whether antegrade or retrograde mesenteric bypass has better outcomes and whether restoration of flow to a single vessel versus multiple mesenteric vessels should be performed. This study reports on our experience using an antegrade prosthetic bypass graft to treat chronic occlusions of the celiac trunk, superior mesenteric artery, and inferior mesenteric artery at their origins, all of which result in visceral ischemia.

## INTRODUCTION

The literature shows that the prevalence of mesenteric artery stenosis is increasing with age [1, 2]. Without revascularization of the intestine, chronic mesenteric ischemia (CMI) can ultimately progress to acute ischemia, resulting in bowel necrosis with high morbidity and mortality rates [3, 4]. However, the best management strategy for CMI is not well-established. Elective intervention by open surgical repair/bypass or endovascular angioplasty has been shown to relieve symptoms, reverse weight loss and prevent progression [5]. Open surgical revascularization (OSR) of mesenteric arteries has been the gold standard since 1958, and is associated with excellent long-term patency and symptom relief [6–8]. However, different approaches were suggested with varying levels of invasiveness and effectiveness. There are ongoing discussions as to whether flow restoration to a single or multiple visceral vessels should be performed and whether an antegrade or a retrograde mesenteric bypass leads to better outcomes. We report a case in which an antegrade bypass of the three mesenteric vessels from the supraceliac aorta was used to manage CMI. Informed and written consents were obtained from patients to publish this report.

## CASE REPORT

**Figure 1:**
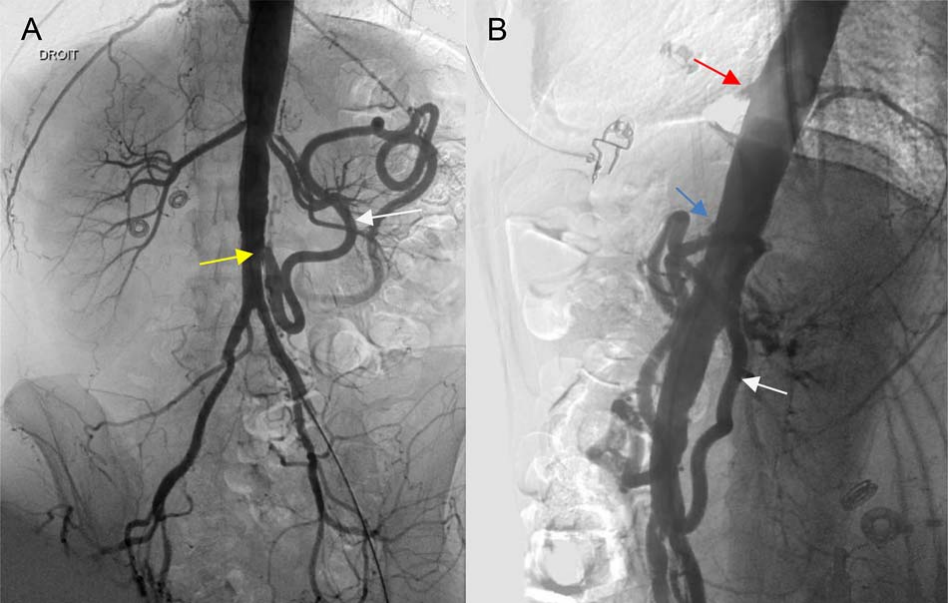
(**A**, **B**) Angiography of the abdominal aorta (preoperative), demonstrating the occlusion of the CT (red arrow) and SMA (blue arrow) with severe stenosis of the IMA (yellow arrow). Retrograde flow of the arc of the Riolan artery is shown (white arrow).

A 46-year-old woman presented with a history of abdominal angina, nausea and vomiting, and weighed 40.9 kg (body mass index 16.4). A computed tomographic angiography (CTA) was used as the imaging modality to evaluate the patient’s symptoms, which revealed occlusion of the celiac trunk (CT), superior mesenteric artery (SMA) and severe stenosis of the inferior mesenteric artery (IMA) at its origin. Mesenteric circulation was supplied via retrograde flow through the arc of the Riolan artery (Fig. 1A, B), with branches of the celiac artery patent at their origin. The two internal iliacs were patent, although both external iliacs were calcified. Coronary angiogram and cardiac echo were unremarkable. The patient underwent a repeated, extensive screening workup for hypercoagulable disorders and vasculitis, which turned out to be normal. The case was presented at a multidisciplinary meeting to evaluate revascularization options; we opted for an open reconstruction based on our experience, available literature reports, and the age and adequate cardiopulmonary reserve of the patient.

To optimize the patient’s nutritional well-being, an endovascular revascularization of the SMA and IMA, was initially attempted as a bridge to open definitive revascularization; however, it was unsuccessful. Thus, the decision was made to proceed with OSR, while median laparotomy was performed with a transperitoneal approach. Abdominal exploration was also performed, including an evaluation of other intra-abdominal pathology and an inspection of the small bowel for unsuspected ischemic perforations. The intestines were found to have minimal ischemic changes. The left liver lobe was mobilized and the hepatic artery was exposed. The SMA was exposed after dissection of the ligament of Treitz and mobilization of the fourth portion of the duodenum below the pancreas. We opted to perform an anterograde revascularization of the CT and SMA from the aorta with an 8-mm polytetrafluoroethylene graft, as well as reimplantation of the IMA to improve the graft’s long-term patency and symptoms: heparin was also administered. To avoid interruption of the blood supply to the kidney, we placed a partial occlusion clamp laterally at the level of the CT. Arteriotomy was performed as well, and the graft was anastomosed end-to-side to the aorta in a non-diseased segment, while the CT was reimplanted at the lateral side of the graft. The proximal SMA was ligated, with the distal end reimplanted at the end of the graft with an end-to-end anastomosis with running 6-0 Prolene sutures. Orientation and tunneling of the graft under the pancreas was also performed. The proximal IMA was ligated, and the distal end was reimplanted within the infrarenal aorta in a disease-free zone. Intestinal color improved immediately after the bypass, with total circulation reestablished as well as satisfactory hemostasis. A standard closure of the laparotomy was performed. The patient was admitted to the intensive care unit as standard protocol for 24 h, making an uneventful recovery; she was discharged on the 10th postoperative day.

During this time, the patient showed no symptoms of renal failure, cerebrovascular accident, myocardial infarction, ventricular arrhythmias, or congestive heart failure. The patient had been on antiplatelet therapy preoperatively, which was resumed on postoperative day 1.

**Figure 2:**
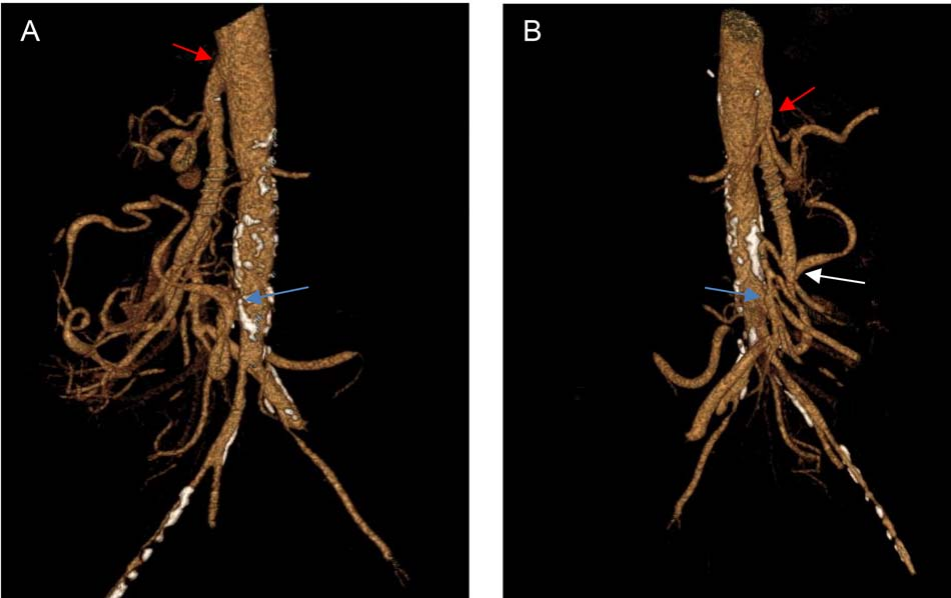
(**A**, **B**) Three-dimensional CT reconstruction at 1-year follow-up, showing the anastomosis of the CT (red arrow), the anastomosis of the SMA (white arrow) and the reimplantation of the IMA (yellow arrow) with excellent graft patency.

The first postoperative follow-up was 2 weeks after surgery, while abdominal CTA scans with 3D reconstruction were performed at 6 months and 1 year (Fig. 2A, B), showing patency of the graft with no anastomotic stenosis or thrombosis. The patient was asymptomatic, gaining 10 kg at 6 months, and did not require open surgical intervention or secondary endovascular procedures during follow-up.

## DISCUSSION

Mesenteric circulation includes the CT, SMA and IMA connected by numerous collateral vessels. Symptomatic CMI demonstrates severe stenosis or occlusion of the SMA alone, with involvement of the CT and IMA. The evolution of endovascular therapies for CMI has reduced the overall number of open procedures, although primary patency, restenosis and symptom recurrence rates with an endovascular approach are traditionally inferior to OSR [10]. Therefore, mesenteric bypass continues to have an important role in the treatment of CMI.

Current OSR involves mesenteric revascularization by endarterectomy, bypass or a combination of both [5]. Mesenteric inflow has traditionally been performed from the distal thoracic aorta, proximal abdominal aorta (antegrade fashion) in our case, or from the distal abdominal aorta or iliac artery (retrograde fashion), using a vein or synthetic graft conduit [9]. However, controversy remains about whether antegrade or retrograde mesenteric bypass has better outcomes and whether flow restoration to a single, versus multiple mesenteric vessels, should be performed. However, Hollier *et al.* [11] found a lower rate of recurrent symptoms with complete antegrade revascularization of the mesenteric vessel, and also found a higher incidence of recurrent symptoms with single-vessel revascularization.

Our patient remained asymptomatic at the 1 year follow-up, improving long-term graft patency and symptoms; an antegrade bypass was performed with total mesenteric vessel revascularization, and the IMA.

Other studies suggest that an antegrade approach with revascularization of all stenotic mesenteric vessels provide the best long-term results [11, 12]. However, van Dijk *et al.* and other studies argue that revascularization of only the SMA is sufficient for successful treatment of CMI [13, 14].

Our choice of anterograde revascularization (versus retrograde) represents a less angulated, shorter bypass of mesenteric vessels. A Mayo Clinic study reports that this is the preferred approach for lower-risk patients, similar to our case, who have multivessel disease with low risk of renal ischemia [10]. In our opinion, the risk of renal malperfusion is decreased by placing a lateral aortic clamp, which has been well-tolerated.

Retrograde revascularization is less invasive, but must be done carefully to avoid graft kinking and angulation following a longer bypass [10]. No data in the literature on acute mesenteric ischemia, bowel gangrene or contamination suggest that an artificial graft has superior patency to an autologous vein graft in mesenteric revascularization. As such, we favor the use of artificial grafts to save the vein for future bypasses, if needed.

## CONCLUSION

In selected patients at low risk for aortic surgeries, as well as younger patients with a relatively disease-free supraceliac aorta, an open antegrade revascularization of all stenotic vessels is a viable treatment approach for the management of CMI.

## AUTHORS’ CONTRIBUTIONS

As per the guidelines of the International Committee of Medical Journal Editors (ICMJE), all authors made substantive intellectual contributions to the study, collaboratively designing, analyzing, and interpreting the research, and writing and revising the manuscript. All authors have given approval for this manuscript to be published.

## ETHICS APPROVAL AND CONSENT TO PARTICIPATE

All procedures performed in studies involving human participants were in accordance with the ethical standards of the institutional and/or national research committee and with the 1964 Declaration of Helsinki and its later amendments or comparable ethical standards.
